# Study on Synergistic Antioxidant Effect of Typical Functional Components of Hydroethanolic Leaf Extract from Ginkgo Biloba In Vitro

**DOI:** 10.3390/molecules27020439

**Published:** 2022-01-10

**Authors:** Lihu Zhang, Chunyi Zhu, Xiaoqing Liu, Erzheng Su, Fuliang Cao, Linguo Zhao

**Affiliations:** 1 College of Chemical Engineering, Nanjing Forestry University, Nanjing 210037, China; zlh800927@163.com (L.Z.); chunyi_zhu@163.com (C.Z.); xiaoqingliu_njfu@163.com (X.L.); 2 Department of Pharmacy, Jiangsu Vocational College of Medicine, Yancheng 224005, China; 3 College of Light Industry Science and Engineering, Nanjing Forestry University, Nanjing 210037, China; ezhsu@njfu.edu.cn; 4 Co-Innovation Center for Sustainable Forestry in Southern China, Nanjing Forestry University, Nanjing 210037, China; caofl1953@163.com

**Keywords:** Ginkgo biloba extract (EGb), synergism, antioxidation, ginkgo flavone, ginkgolide

## Abstract

The predicted anti-oxidation is related to apoptosis, proliferation, lipid metabolism, cell differentiation, and immune response. There are some differences in the antioxidant capacity of the four typical components of ginkgo biloba extract (EGb) including ginkgo flavone (GF), ginkgolide (G), procyanidins (OPC), and organic acids (OA), and any two members of them can exhibit apparent synergistic effects. The order of DPPH scavenging ability was: OPC > GF > OA > G. The scavenging ability of procyanidins was close to that of VC; the scavenging capacity of ABTS was GF > OPC > OA > G. The GF:OPC (1:9) showed the best synergism in scavenging DPPH and ABTS radicals. The 193 kinds of small molecules reported in EGb were obtained by analyzing the properties of EGb. In order to construct a corresponding biological activity target set, molecular docking and the network pharmacology method were employed to build the molecular action mechanism network of a compound target, and the main biological functions and signaling pathways involved with their antioxidant activities were predicted. The results displayed that the top ten compounds which belonged to the two broad categories, ginkgo flavonoids and proanthocyanidins, could interact closely with several important target proteins (CASP3, SOD2, MAPK1, HSPA4, and NQO1). This would be expected to lay a theoretical foundation for the deep development of Ginkgo biloba extract.

## 1. Introduction

At present, Ginkgo biloba extract (EGb) can be regarded as a typical representative of traditional Chinese medicine (TCM), which displays multi-component, multi-target, and multi-activity characteristics [1]. The Schwabe company has given much attention to the various pharmacological effects of EGb [2]. The biological activities of Ginkgo biloba extracts produced by different production processes are different. Although various chemical components of EGb are integrated, there are complementary and synergistic effects between several members. However, one or several active ingredients in traditional Chinese medicine cannot represent the overall efficacy. In addition, some enterprises at home and abroad pursue high ketone esters, such as the Shanghai Xingling Company, which produces raw materials for the new Class II ginkgo ketone esters: flavonoid glycosides ≥ 44% and lactones ≥ 6%, which is far from the “24 + 6” standard of EGb761^3^. The high ketone ester would change the content of the ingredients. In other words, 24% of flavonoids and 6% of lactones are the main active ingredients of Ginkgo biloba extract. Is it necessary to add the other 70% of the ingredients? Whether there is a synergistic effect between the effective factors deserves further study [3].

During cellular metabolism, reactive oxygen species (ROS) are produced, leading to cellular damage and disease. It mainly includes superoxide anion, NO radicals, hydroxyl radicals, and hydrogen peroxide. It has been found that the occurrence and development of many diseases are closely related to the oxidation of oxygen free radicals, for example, cardiovascular diseases, neurodegenerative diseases, atherosclerosis, tumors, etc. [4,5,6,7]. Ginkgo biloba extract can prevent and inhibit the toxicity of oxygen free radicals and reduce lipid-induced peroxidative damage. In EGb, ginkgo flavonoids, proanthocyanidins, and organic acids have a large number of reduced hydroxyl functional groups, which can play an antioxidant role by scavenging oxygen free radicals and regulating the activity of superoxide dismutase and catalase.

According to various animal model studies, EGb exhibited obvious antioxidant functions. EGb plays a role mainly directly inactivating various oxygen free radicals, increasing the activity of antioxidant enzymes such as glutathione peroxidase (GSH-Px), and inhibiting lipid peroxidation [8], and has strong biological effects such as antioxidation, scavenging free radicals activity [9]. Wang et al. [10] reported that the study of EGb exhibited the effects of increasing superoxide dismutase (SOD) activity and decreasing the apoptosis rate of diabetic vascular cells in a dose-dependent manner. Meanwhile, the animal model study revealed that EGb can reduce oxidative stress and significantly increase antioxidant capacity in diabetic rat hearts [11]. Ischemia-reperfusion injury is the most obvious damage to the antioxidant system of the animal organisms, and the application of antioxidants is very important to reduce oxidative damage. The protective effect of EGb on ischemia-reperfusion injury of myocardial and neural tissues has been demonstrated, the protective mechanism of which is that EGb can directly inactivate various oxygen radicals, inhibit lipid peroxidation reactions, and increase antioxidant enzymes such as SOD and GSH-Px [12,13]. Besides, Wang et al. [14] found that EGb exhibited antioxidant and radical scavenging effects in an animal model of acute liver injury, involving reducing the production of free radicals and increasing GSH-Px and SOD activities. Xiao et al. [15] found that EGb had significant protective effects against oxidative stress injury in melanocytes in a dose-dependent manner.

Ginkgo biloba extract, as a kind of traditional Chinese medicine with multi-component, has four typical components: ginkgo flavone (GF), ginkgolide (G), procyanidins (OPC), and organic acids (OA). Its antioxidant activity has been associated with the complex network regulatory mechanism. In this study, to investigate the antioxidant activity of each component and the synergistic antioxidant effects between different components, we would attempt to compare the antioxidant activity of each component based on two classical anti-oxidation tests firstly. Secondly, the combinations of various components in different proportions will be explored, and the best match between different components could be revealed. In addition, The study of network pharmacology can be employed for predicting new targets, establishing molecular target-disease network relationships, and analyzing their antioxidant mechanisms more effectively [16,17,18]. The results from the network pharmacology study will also be used for explaining the antioxidant activity of EGb components, particularly GF, G, OPC, and OA.

## 2. Results and Discussion

### 2.1. Preparation of Four Main Components from EGb

The two components (Ginkgo flavone and ginkgolide) were prepared from EGb, while proanthocyanidins and organic acids were extracted from the corresponding Ginkgo biloba leaves by deep eutectic solvents. To ensure the accuracy and quality of the prepared samples and provide reliable test samples for the study of the activity and synergistic relationship of the typical effective components of EGb, the related samples have been treated several times according to the same method above. The obtained results verified that the contents of these components seemed to show little change (HPLC spectrum (Figures S1 and S2 in Supplementary Materials).

### 2.2. DPPH Free Radical Scavenging Ability of Typical EGb Components

**Figure 1:**
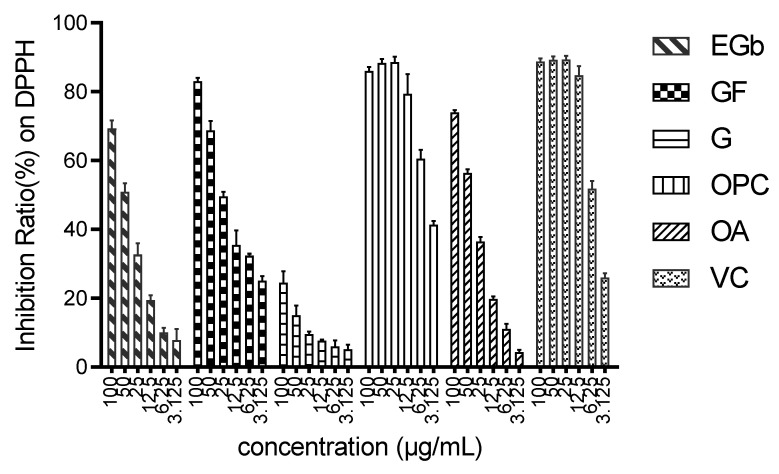
Scavenging effects of EGb functional components on DPPH free radicals.

DPPH scavenging ability is one of the common methods to evaluate the antioxidant activity of compounds. DPPH is a stable solid free radical with an absorption wavelength of 517 nm. When the free radical is removed, the color changes from purple to light or colorless. The antioxidant capacity of the compound is evaluated by measuring the OD value. To investigate the antioxidant capacity of Ginkgo biloba extract (EGb), four common components, ginkgo flavone (GF), ginkgolide (G), procyanidins (OPC), and organic acids (OA) in EGb, together with VC as the positive control, were selected and studied as antioxidant agents. The IC_50_ values of these components in scavenging DPPH free radicals are displayed in Table 1. Furthermore, the scavenging capacity at different concentrations are illustrated in Figure 1.

The results showed that procyanidins, ginkgo flavones, and organic acids exhibited strong scavenging capacity, respectively, while ginkgolide seemed to show a weak activity (IC_50_: 672.2 μg/mL). The antioxidant activity of proanthocyanidins should be closely related to its structure. Because the molecular structure of proanthocyanidins contained several active phenolic hydroxyl groups, which can provide hydrogen to free radicals, it exhibited potent activity. Moreover, it was seen that such compounds could be able to scavenge DPPH free radicals and were also dose-dependent when compared with vitamin C. Ginkgo flavones also had phenolic hydroxyl groups, and however, the capacity of these compounds was slightly inferior to that of procyanidins. In addition, the lack of hydroxyl groups in the chemical structure of ginkgolides led to the loss of scavenging free radicals.

### 2.3. The Synergistic Scavenging Effect of the Typical Components of EGb on DPPH Radical

The typical effective components of EGb were compounded in pairs, and their ability to scavenge DPPH free radicals was investigated. The IC_50_ values of these compounds with different proportions were calculated by GraphPad software, as shown in Table 2.

Five different proportions of the two components from EGb four components were mixed and then tested for their synergistic antioxidant effects. The obtained results in Table 2 displayed that most of the combinations including ginkgolide exhibited an antagonistic effect on scavenging DPPH free radicals. Interestingly, the combination of proanthocyanidins and Ginkgo flavones in the ratio of 1:9 showed the most potent synergistic effect (interaction coefficient γ: 0.5), while this combination possessed an antagonistic effect at the other four compounding ratios.

### 2.4. ABTS Method Was Employed for Determining the Antioxidant Capacity of the Main Effective Components of EGb

**Figure 2:**
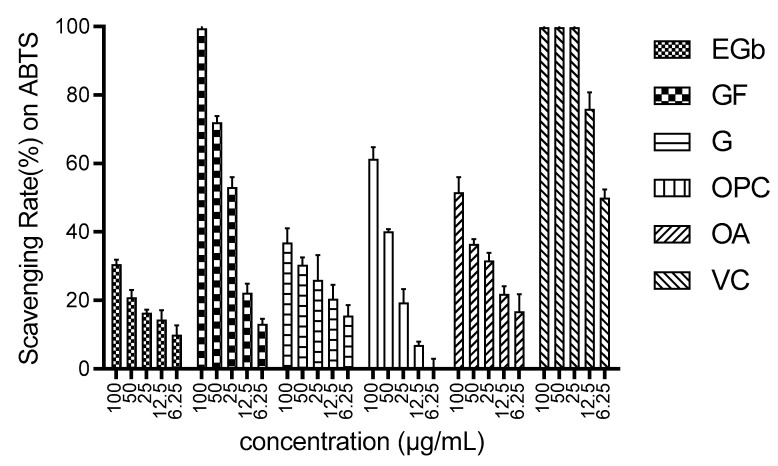
Scavenging effect of EGb functional components on ABTS free radicals.

K_2_S_2_O_4_ can oxidize the colorless ABTS to form a stable blue-green ABTS+ free radical, combining with antioxidants to change back to colorless ABTS. Additionally, ABTS+ has the strongest absorption at 734 nm. The ability of the tested samples to remove ABTS+ could be examined by comparison of the absorbance change at 734 nm. The IC_50_ values of typical effective components of EGb, the positive control vitamin C, and their scavenging capacity at different concentrations are displayed in Figure 2 and Table 3, respectively. As the same trend with DPPH tests, the most potent component was GF, the IC_50_ value of which was 24.25 μg/mL, far superior to the other three components. Surprisingly, however, the antioxidant activity of EGb seemed very poor (IC_50_: 204.4 μg/mL). The antagonistic effect between these components would be one of the potential causes.

### 2.5. Isoradiation Method Was Employed for Analyzing the Synergistic Effect of EGb on ABTS Free Radical

As mentioned before, four kinds of typical EGb components were compounded, and the five combination ratios (9:1, 7:3, 1:1, 3:7, and 1:9, respectively) were investigated. The clearance rate of ABTS at different concentrations was determined for each compound, and meanwhile, the corresponding IC_50_ value at each proportion was calculated. The obtained data were further processed by the isoradiometric method. The interaction coefficients (γ) are displayed in Table 4.

Unlike the DPPH assay, most combinations of OPC and GF can exhibit a potent synergistic effect on the scavenging of ABTS free radicals. As to the two components, the best combination was still OPC and GF. The most potent synergistic coefficient (γ) was 0.39 when the ratio between OPC and GF was 1:9. The other combinations such as G + GF, OA + GF, G + OPC, and OA + G could not produce obvious synergistic effects.

### 2.6. Analysis of EGb Molecular Descriptors

The molecular descriptors of EGb components were statistically analyzed. The results of maximum, minimum, average, and median values are shown in Table 5.

**Figure 3:**
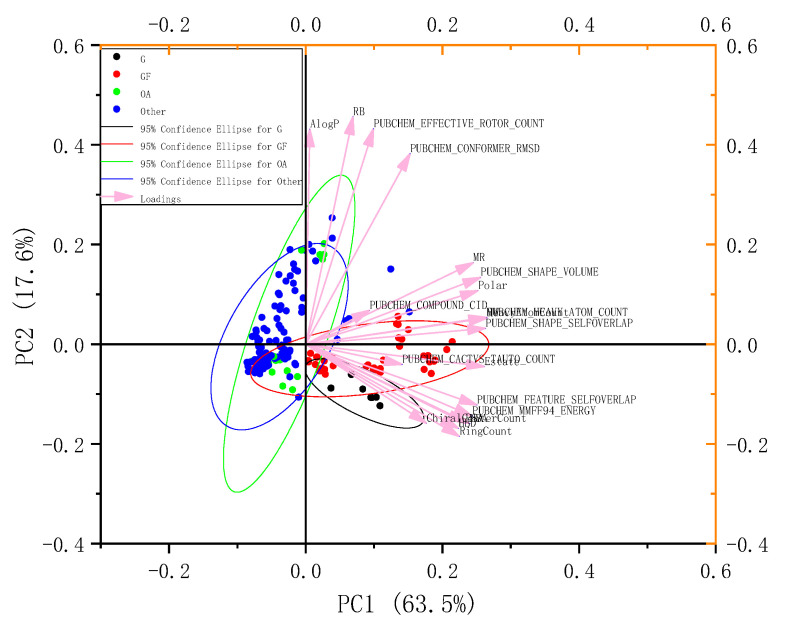
Principal component analysis of the physical and chemical properties of the 193 small molecules belonging to EGb.

The results showed that 193 of the 467 compounds were drug-like, implying that EGb had good oral absorption properties. The average polar surface area ratio of EGb was 64.97, and the average number of rotatable bonds was 4.56 (median 2.00), which also indicated that most of the molecules in EGb had good bioavailability (≥20%). In addition, these ten relatively independent descriptors (Table 5) had been discussed based on the principal component analysis method. The main component analysis (PCA) results of 193 compounds are shown in Figure 3.

The PC1 component can account for 63.5% and the second component (PC2) accounts for 17.6. It can be seen from Figure 3 that two classes of EGb compounds, G and GF, had a large overlap in the PC1 main descriptor, while OA and other compounds have a partial overlap in the PC2 main descriptor. Since the PC1 contained several relative molecular descriptors, such as molar refractive index (MR), molecular polar surface area ratio (Polar), etc., the two kinds of EGb compounds (G and GF) might be similar biological activities. Interestingly, there indeed was a synergistic relationship between the two components in terms of antioxidant activities.

### 2.7. Construction and Characteristic Analysis of Molecular Antioxidant Network

The crystal structures of human antioxidant target proteins were searched from the PDB protein database of RSCB. After the manual curated screening, there were 27 antioxidant targets in total (Table 6).

**Figure 4:**
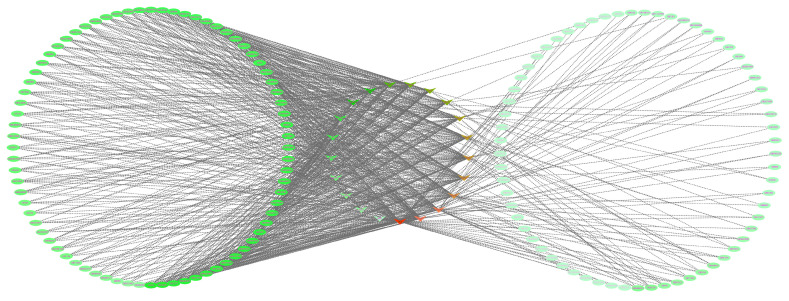
Network chart of compound-target protein in compounds in EGb.

Based on the molecular docking study between 193 EGb small molecules and 27 antioxidant-related target proteins, the “drug-target” network relationship between the EGb molecules and the antioxidant target was constructed according to the score of molecular docking. The software Cytoscape 2.8.1 was employed for the construction of the network. The corresponding network can be seen in Figure 4.

As shown in Figure 4, the overall characteristics of the EGb molecular antioxidant target network were analyzed. The results showed that the number of nodes was 163, the average number of adjacent nodes was 9.975, the shortest path was 26406 (100%), the characteristic path length was 2.388, the network density was 0.062, the network centrality was 0.625, and the network heterogeneity was 1.510. EGb molecules and antioxidant targets have the characteristics of multi-component and multi-target synergistic effects. By selecting the top ten antioxidant target proteins and EGb molecules in the network to understand the synergistic effect in the biological network, the network characteristic analysis values are shown in Table 7.

It can be seen from Table 3 that 113 EGb molecules have interaction with ALB target protein. It was noted that ALB target protein has the best network degree in the EGb molecule antioxidant target protein network. The other target proteins are CASP3, SOD2, MAPK1, HSPA4, NQO1, G6PD, GSTK1, and KEAP1. However, the top ten compounds with a good EGb molecular interaction network are ginkgo flavonoids, proanthocyanidins, and organic acid 6-hydroxykynurenic acid (6-HKA).

### 2.8. Characteristic Analysis of Antioxidant Target Protein Pathway Network

**Figure 5:**
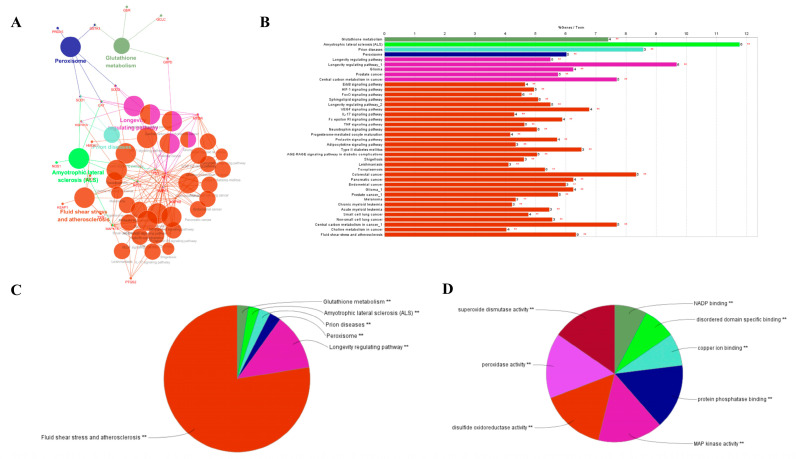
Molecular function and metabolic pathway analysis of EGb. (**A**) “Targets-Pathway-Disease” network of EGb; (**B**) Gene percent involved with these pathways and diseases; (**C**) Six primary pathways related with antioxidation; (**D**) Eight antioxidant mechanisms of EGb. ** Indicates that the *p*-value is less than 0.01.

Enrichment analysis of antioxidant metabolic pathways can aggregate similar biological targets to form functional modules. The Cluego plug-in analyzed the biological function and metabolic pathway of its antioxidant target. The results are shown in Figure 5.

The biological functions of Ginkgo biloba extract on antioxidant targets are mainly concentrated in eight aspects, including superoxide dismutase activity, nicotinamide adenine dinucleotide phosphate (NADP) binding, disordered domain-specific binding, copper binding, protein phosphatase binding, the activities of phosphotase binding, mitogen activated protein (MAP) kinase activity [20], disulfide oxidoreductase activity, and peroxidase activity were measured. The signal pathways of EGb acting on antioxidant targets are mainly enriched in six aspects: fluid shear stress and atherosclerosis signaling pathway, glutathione metabolism signaling pathway, and amyotrophic lateral sclerosis signaling pathway. The results showed that there were three pathways, including the prion diseases signaling pathway, peroxisome signaling pathway, and longevity regulating pathway [21].

## 3. Conclusions

The obtained results showed that the antioxidant activities of the four typical components in EGb were different and synergistic, which was similar to the results of the previous antioxidant prediction. The order of DPPH scavenging capacity was OPC > GF > OA > G, and the procyanidins scavenging ability was close to that of VC; the ability to scavenge ABTS GF > OPC > OA > G was lower than that of VC positive control. At the same time, it was found that organic acids also had a synergistic effect on the antioxidant activity of Ginkgo flavone. The 193 compounds with good bioavailability were obtained by a drug-like analysis and chemical spatial principal component analysis on the molecular data set constructed by EGb. Principal component analysis showed that Ginkgo flavone and ginkgolides overlapped in chemical space, and organic acids and flavonoids partly overlapped, which indicated that the molecular descriptors such as polarity, lipid water partition coefficient, and so on, were similar, and there might be a synergistic relationship between the components in biological activities. In addition, APK1, GK2, and APK4 are the targets of APK1, GK113, and other proteins. However, the compounds with a good network degree are all Ginkgo flavones, proanthocyanidins, and organic acid 6-HKA, which indicates that there is a synergistic relationship among Ginkgo flavones proanthocyanidins and organic acids [22,23,24]. It was found that the antioxidant activity of Ginkgo biloba extract was concentrated in 8 biological functions and six signal pathways. This paper confirmed that there is a synergistic effect between EGb components, but the mechanism of synergism needs to be confirmed by further molecular biology experiments.

## 4. Experimental Materials and Methods

### 4.1. Experimental Reagents and Instruments

The solvents (methanol and ethanol) were purchased from Nanjing Jianghua Glass Instrument Co., Ltd. (Nanjing, China). The DPPH (2, 2-Di(4-tert-octyl phenyl)-1-picrylhydrazyl, free radical) was purchased from Sigma. The ABTS (2’-Azinobis-(3-ethylbenzthiazoline-6-sulphonate) reagent was purchased from Shanghai Biyuntian Biotechnology Co., Ltd. (Shanghai, China). All the instruments and equipment were listed as follows: HH-4 warm water bath (Guohua Electric Co., Ltd., Nanjing, China); IKARV05 basic rotary evaporator (IKA group, Germany); KH3200B ultrasonic cleaner (Kunshan Hechuang Ultrasonic Instrument Co., Ltd., Suzhou, China); SBH-III circulating water multi-purpose vacuum pump (Zhengzhou Great Wall Technology Industry and Trade Co., Ltd., Zhengzhou, China); WH861 vortex mixer (Taicang science and education equipment factory); BS124s Electronic balance (Beijing saiduolis Instrument System Co., Ltd., Beijing, China); Allegra x-22r desktop high-speed centrifuge (Beckman Kurt Co., Ltd., Brea, CA, USA); QB-9006 constant temperature microporous plate fast oscillator (Shanghai Shupei Experimental Equipment Co., Ltd., Shanghai, China); direct-plate electrophoresis instrument and film transfer instrument (Bio Rad Co., Ltd., Hercules, CA, USA); TY-80R Decoloring shaker (Nanjing Kai Ji Biotechnology Co., Ltd., Nanjing, China); UVP gel imaging system (Beckman Kurt Co., Ltd., Brea, CA, USA); vertical flat electrophoresis apparatus (Bio-Rad, USA); and UPLCLTQ-Orbitrap XL (Thermo Co., Ltd., Hercules, CA, USA).

### 4.2. Preparation and HPLC Analysis of the Typical Efficacy Components of EGb

A mixture of 20 g of dried EGB was taken into diluted ethanol (70%, volume ratio, 200 mL), stirred, and cooled to 12 °C. The filtrate was extracted three times with 9:1 ethyl acetate-hexane, and each time with 1/3 of the filtrate volume. The organic phase was washed twice with water, and each time with 1/5 of the organic phase volume. The solid mass of activated carbon (4×) was added, stirred, and adsorbed for 1 h, and subsequently, it was filtered, separated, and washed with a small amount of ethyl acetate. The ginkgo lactone sample can be prepared by concentrating filtrate and washing liquid under reduced pressure. After extraction, a small amount of dissolved organic phase was removed. The organic phase was washed with water after three extraction times with 1/3 volume of n-butanol. The organic solvent was completely removed by vacuum concentration. The residue was a sample of ginkgo flavonoids. Besides, proanthocyanidins [25] and organic acids were prepared from DESs [26,27,28,29]. The components were weighed and incubated at a concentration of 1 mg/mL and then diluted to a concentration of 20–50 μg/mL of the substance to be measured. The four functional components were sampled simultaneously to prepare HPLC or HPLC-ELSD test samples.

### 4.3. Determination of DPPH Clearance Rate

Then, 1.927 mg of DPPH were accurately weighed and then dissolved in 10 mL of methanol at a concentration of 0.5 mmol/mL and stored at 0–4 °C away from light. The sample to be tested was obtained by EGb gradient dilution, and 100 μL of the sample were put into the enzyme standard plate. Subsequently, 100 μL of DPPH solution were added, and the reaction was carried out in the dark at room temperature for 30 min, and finally, the absorbance at OD517 nm was measured. The *A*_2_ represented a normal group that did not contain the sample to be tested, and the *A*_0_ represented the blank group without DPPH. The clearance rate was calculated according to the following formula. Each sample has three parallel samples.

$$\mathrm{DPPH}\;\mathrm{clearance}\%=\frac{A_{2}-\left(A_{1}-A_{0}\right)}{A_{2}}\times 100$$

where: *A*_0_ is the blank absorbance of the determination solution

*A*_1_ is the absorbance after adding the sample to be measured

*A*_2_ is the absorbance without a sample to be tested

### 4.4. Determination of Total Antioxidant Capacity by ABTS Method

The total antioxidant capacity was detected by the ABTS method according to the instructions of the ABTS kit. The ABTS mother liquor was prepared according to the number of samples to be tested. The ABTS solution and oxidant solution were evenly mixed at room temperature and stored in the dark for 12–16 h. The ABTS mother liquor was diluted with 80% ethanol before use. The sample of certain concentration was mixed with 2 mL of methanol, centrifuged, and diluted gradiently to obtain the sample to be tested. Next, 200 μL of ABTS working solution were added into each hole of the enzyme label plate, and then 10 μL of the sample to be tested were added. After being incubated in the dark at room temperature for 5 min, the absorbance A_1_ at 734 nm wavelength was determined. The calculation method of the ABTS^+^·scavenging rate is the same as that of DPPH radical scavenging.

### 4.5. Isoradiometric Analysis

**Figure 6:**
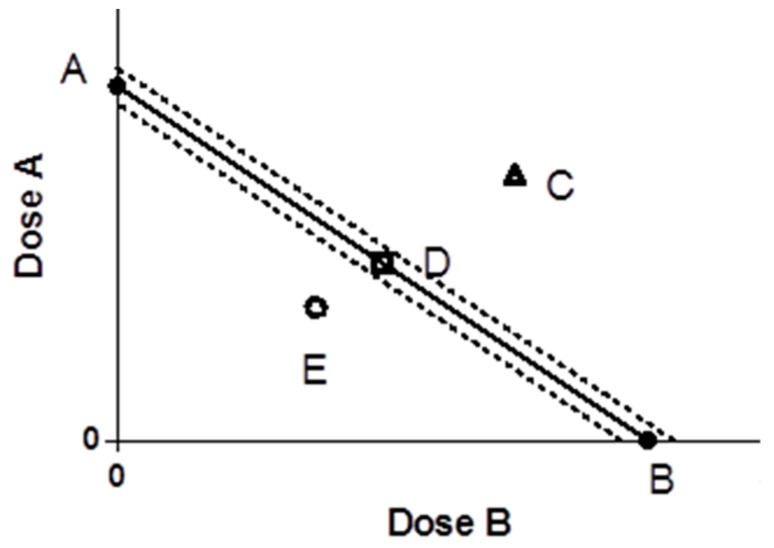
Isoradiation analysis of drugs A and B.

The four typical components of EGb were prepared as 10 mg/mL mother liquor, and the fixed ratio (volume ratio) of them was 9:1, 7:3, 1:1, 3:7, and 1:9. Then, 18 μL of Ginkgo flavone and 2 μL of ginkgolide were added to 200 μL volume to obtain the concentration of 100 μg/mL GF and G (volume ratio of 9:1). The DPPH scavenging rate and ABTS clearance rate of the abovementioned mixed samples with different proportions were determined. The IC_50_ value was calculated by GraphPad 9.0 software. The IC_50_ value of drug A was used as ordinate, and the IC_50_ value of drug B was used as abscissa. The IC_50_ value of compound A was ordinate and the IC_50_ of compound B was abscissa. Coordinate position points were determined and three functions were judged. Evaluation of the degree of synergistic or antagonistic action is often expressed by interaction coefficient (γ) (Figure 6) [30].

A: The dose of drug A when used alone; B: the dose of drug B when used alone; the line segment connecting the two points of AB is the contour line; the dotted line is the 95% confidence line. C is synergistic, E is antagonistic, and D is additive.

$$\gamma =\frac{{\mathrm{IC}}_{50\mathrm{Amix}}}{{\mathrm{IC}}_{50A}}+\frac{{\mathrm{IC}}_{50\mathrm{Bmix}}}{{\mathrm{IC}}_{50B}}$$

IC_50Amix_ and IC_50Bmix_ were the IC_50_ values of A and B antioxidants in the compound group; IC_50A_ and IC_50B_ were the IC_50_ values of A and B antioxidants alone. If γ = 1, it indicates that the interaction is additive; if γ < 1, it indicates that the interaction is synergistic, and the more obvious the value of γ, the stronger the synergistic effect; if γ > 1, it indicates that the interaction is antagonistic [31].

### 4.6. Data Collection and Structure Processing of EGb Small Molecular Compounds

Using the TCM information database TCMSP (http://ibts.hkbu.edu.hk/LSP/tcmsp.php, The last visit was on 11 October 2020) and National Center for Biotechnology Information (NCBI) database (https://www.ncbi.nlm.nih.gov/, The last visit was on 11 October 2020.), 465 compounds with different structures were collected after optimization analysis. At the same time, the corresponding compounds were downloaded from the PubChem database and saved in SDF format. By importing these molecules into Maestro 10.1, using the living preparation module for small molecule processing, selecting the opls 2005 force field default settings for conformational optimization, and then importing the prepared ligand molecules into canvas in Schrodinger 2015 software 2.3, the module calculates the related molecular descriptors (relative molecular weight, number of rotatable bonds, number of hydrogen bond acceptors, number of hydrogen bond donors, number of molecular rings, number of molecular aromatic rings, molecular volume, molecular surface area, molecular polar surface area, lipid water partition coefficient). According to Lipinski’s “five principles of drug-like” [32], the main parameters are relative molecular weight <500, the number of hydrogen bond donors <5, the number of hydrogen bond receptors <10, the partition coefficient of lipid and water <5, and the number of rotatable bonds not more than 10.

### 4.7. Principal Component Analysis of EGb Molecular Data Set

Import the EGb molecular data set into canvas 2.3, calculate the corresponding molecular descriptors, and select 22 of the 60 important molecular descriptors as Alogp, Chiralcentercount, Chiral-center-count, Estate, HBAS, HBD, Heavy atom count, MR, MW, PSA, Cactvs-Tauto-Count, Compound-CID, Conformer-RMSD, Effective-rotor-count, Feature-Selfoverlap, Heavy-Atom-Count, Polar, RB, MMFF94-Energy, Shape-Selfoverlap, SHAPE. The EGb compounds were divided into four categories: Ginkgo flavone, ginkgolide, organic acid, and other components. The PCA plug-in of Origin 8.0 was employed for analysis, and the results were transformed into a 2D and 3D chemical spatial map of Ginkgo biloba extract molecules.

### 4.8. Screening of Antioxidant Related Targets

The target proteins of anti-oxidation, anti-inflammatory and anti-tumor approved by the FDA were selected from the Drugbank database [33], TTD database [34], and COOLGEN database, and the representative targets of anti-oxidation were selected based on the existing research results.

### 4.9. Molecular Docking Process

The 193 molecules reported in EGb and the existing inhibitor molecules in the anti-inflammatory target protein crystal were introduced into Maestro 10.1 and prepared by the living preparation module. The current inhibitor molecules in anti-inflammatory target protein crystal do not perform energy minimization but only deal with hydrogenation, distribution elements, and bond types. The human receptor protein structure was downloaded from the RSCB protein database and imported into the protein preparation module of Maestro 10.1. The target proteins were modified, dehydrated, and hydrogenated under the default parameters, especially the eutectic water molecules around the ligand molecules in the protein crystal (<5 × 10^−10^ m), and the ligand molecules themselves were removed from the docking lattice, and the tautomeric structure and ionic residues were standardized and optimized in the pH neutral state. Finally, 34 kinds of receptor target proteins and 193 kinds of ligand compounds optimized by the software were introduced into Glide 6.6 software for molecular docking simulation.

### 4.10. Docking Process

Standard precision (SP) was set for docking 193 EGb molecules, and the conformations of the existing inhibitors in the protein crystal structure were not searched. The sampling process was set to “none (score in place only)”, and the scoring operation was only performed. The active site is defined by the location of the existing inhibitors in the protein crystal. The lattice box of the active site is set as: x = 2 × 10^−9^ m, y = 2 × 10^−9^ m, z = 2 × 10^−9^ m, and others are default settings. Monte Carlo (MC) algorithm was used to search for the reasonable conformation of the docking compounds in the active site area, and the docking results were screened. The target protein was butted with the existing ligands in the protein crystal. The docking fraction was used as the reference value; secondly, the docking fraction was better than the reference value, and the results were sorted.

### 4.11. Construction of Molecular Target Protein Interaction Network

A molecular target network of EGb small molecules interacting with anti-inflammatory receptor target proteins was constructed by selecting the top 1000 pairs of molecules and target proteins and importing their information into the software of Cytoscape 2.8.1. Nodes represent EGb ligand molecule and anti-inflammatory receptor target protein, and the relationship between EGb ligand molecule and receptor target protein is defined by an edge. Network analyzer, a plug-in in the software, analyzes the characteristic parameters (network degree, network density, medium number, and shortest path, etc.) [35,36], predicts the potential active molecular groups and potential target protein groups in EGb components, and lists the top 10 receptor target proteins and EGb functional molecular information.

### 4.12. Gene Pathway and Function Analysis

The gene function of the anti-inflammatory target was analyzed by molecular biological function in Cluego, a plug-in of Cytoscape 2.8.1 software. The metabolic pathway of the active target was enriched by Kyoto gene and genome Encyclopedia (KEGG). The species is Homo sapiens, ontology reference set, the kappa score threshold is 0.4, *p*-value ≤ 0.05, and the rest are set as default parameters. The node represents the target and metabolic pathway in the generated network, while the edge represents the interaction between the target and route.

### 4.13. Statistical Analysis Method

In this section, GraphPad Prism 5 software (Version 5.01) was used to analyze the data of antioxidant, anti-inflammatory factors, tumor cell proliferation inhibition rate, gray analysis, etc. The data were expressed as mean ± standard deviation, and one-way ANOVA was used between groups, *p* < 0.05 was the difference, with statistical significance.

## Figures and Tables

**Table 1:** The IC_50_ value of DPPH radical scavenging from each functional component of EGb. (Ginkgo biloba extract (EGb), ginkgo flavone (GF), ginkgolide (G), procyanidins (OPC), and organic acids (OA)).

Component	EGb	GF	G	OPC	OA	VC
IC_50_ (μg/mL)	47.82 ± 1.05	19.56 ± 0.763	672.2 ± 0.627	4.009 ± 0.358	40.40 ± 1.16	5.725 ± 0.612

**Table 2:** Synergistic effect of functional components of EGb scavenging DPPH free radical.

Type of Compound	Proportion	The IC_50_ Value of DPPH Scavenging Effect of Compound (μg/mL)		
		Theoretical IC_50_ Value	Measured IC_50_ Value	γ
G + GF	9:1	155.01	139.0	0.90
	7:3	61.05	104.5	1.71
	1:1	38.01	77.73	2.04
	3:7	27.60	40.42	1.46
	1:9	21.66	53.06	2.45
OPC + GF	9:1	18.84	14.37	3.30
	7:3	9.04	12.85	2.44
	1:1	6.65	14.26	2.14
	3:7	5.26	13.34	1.48
	1:9	4.36	7.095	0.50
OA + GF	9:1	20.37	47.86	2.32
	7:3	23.14	41.4	1.79
	1:1	26.36	36.25	1.38
	3:7	30.61	29.78	0.97
	1:9	36.51	28.25	0.77
G + OPC	9:1	38.05	152.62	4.01
	7:3	13.18	39.57	3.00
	1:1	7.97	26.15	3.28
	3:7	5.71	16.86	2.95
	1:9	4.45	9.66	2.17
OA + G	9:1	262.18	37.64	0.84
	7:3	118.10	45.74	0.81
	1:1	76.22	67.83	0.89
	3:7	56.27	74.46	0.63
	1:9	44.59	237.5	0.91

**Table 3:** The IC_50_ value of ABTS radical scavenging from each functional component of EGb.

Components	EGb	GF	G	OPC	OA	VC
IC_50_ (μg/mL)	204.4 ± 0.137	24.25 ± 1.70	377.6 ± 0.402	69.44 ± 1.433	101.8 ± 0.594	6.414 ± 2.07

**Table 4:** Synergistic effect of functional components of EGb scavenging ABTS free radical.

Type of Compound	Proportion	The IC_50_ Value of ABTS Scavenging Effect of Compound (μg/mL)		
		Theoretical IC_50_ Value	Measured IC_50_ Value	γ
G + GF	9:1	58.53	140.12	0.91
	7:3	44.54	75.56	1.07
	1:1	35.95	56.32	1.24
	3:7	30.13	42.07	1.25
	1:9	25.94	36.02	1.35
OPC + GF	9:1	58.53	74.33	1.27
	7:3	44.54	42.81	0.96
	1:1	35.95	24.26	0.67
	3:7	30.13	16.36	0.54
	1:9	25.94	10.01	0.39
OA + GF	9:1	77.13	87.56	3.34
	7:3	51.96	51.42	1.64
	1:1	39.17	46.25	1.18
	3:7	31.43	49.71	0.96
	1:9	26.25	31.65	0.41
G + OPC	9:1	261.54	256.62	0.98
	7:3	161.97	209.51	1.29
	1:1	117.31	126.15	1.08
	3:7	91.95	104.86	1.14
	1:9	75.61	89.66	1.19
OA + G	9:1	71.72	127.67	1.16
	7:3	76.76	155.46	1.19
	1:1	82.56	148.23	0.92
	3:7	89.31	231.46	1.11
	1:9	97.27	337.5	1.14

**Table 5:** Molecular descriptors of compounds contained by EGb.

Molecular Descriptors	Minimum Value	Maximum Value	Average Value	Median Value
Lipid/water partition coefficient (AlogP)	−2.74	12.95	3.26	2.77
Relative molecular mass	84.12	668.60	424.40	212.29
Number of hydrogen bond receptors	0	17.00	3.54	1.00
Number of hydrogen bond donors	0	10.00	1.88	1.00
Number of rotatable keys	0	28.00	4.56	2.00
Molar refractive index	23.15	193.62	73.15	68.01
Number of molecular aromatic rings	0	1.00	6.00	1.96
Molecular polar surface area ratio (Polar)	0	269.43	64.97	29.46
Number of heavy atoms	6.00	47.00	19.27	16.00
Number of chiral centers	0	14.00	1.90	0

**Table 6:** Known potential proteins as the antioxidant targets.

Target Protein	Full Name	PDB ID [19]
CAT	Catalase	1DGH
SOD1	Superoxide dismutase 1	2WZ0
GSR	Glutathione reductase	2GH5
HMOX1	Heme oxygenase 1	3TGM
SOD2	Superoxide dismutase 2	4A7V
LPA1	Lysophosphatidic acid receptor 1	4Z34
GSTK1	Glutathione S-transferase kappa 1	3RPN
ALB	Serum albumin	4Z69
AChE	Acetylcholinesterase	5HFA
Caspase-3	Caspase-3	3KJF
BCL2	Bcl-2-like protein 1	4QVX
MPO	Myeloperoxidase	4C1M
KEAP1	Kelch-like ECH-associated protein 1	5DAF
MAPK1	Mitogen-activated protein kinase 1	4QTE
AKT1	Threonine-protein kinase	5KCV
NQO1	NAD(P)H dehydrogenase 1	2F1O
G6PD	Glucose-6-phosphate 1-dehydrogenase	2BH9
NOS	Nitric oxide synthase	3HR4
E3Mdm2	E3 ubiquitin-protein ligase Mdm2	5HMK
MTOR	Phosphatidylinositol-4,5-bisphosphate-3-kinase catalytic subunit gamma isoform	3PRE
PTGS2	Prostaglandin G/H synthase 2	5IKT
MAPK8	Mitogen-activated protein kinase 8	4QTD
PRDX5	Peroxiredoxin-5	4MMM
TXN	Thioredoxin	5DQY
HSPA1	Heat shock 70 kDa protein 1	1S3X
MAPK14	Mitogen-activated protein kinase 14	4ZTH
GCLC	Glycogenin-1	3U2V

**Table 7:** Network features of part nodes in the D-T network in EGb.

Node	Betweenness	Network Degree	Node	Betweenness	Network Degree
ALB	0.41420	113	Epicatechin	0.0091845	16
SOD2	0.15260	69	Quercetin	0.0085938	15
MAPK1	0.074029	62	Epigallocatechin	0.0084424	15
HSPA4	0.052319	59	6-HKA	0.0094497	14
NQO1	0.065040	59	Apigenin	0.0089222	14
G6PD	0.044772	56	Ginkgolin	0.0093173	14
GSTK1	0.041743	53	Myricetin	0.0069732	14
KEAP1	0.041642	47	Isorhamnetin	0.0068786	14
CAT	0.043828	47	Catechin	0.0063781	14

## Data Availability

Not applicable.

## Supplementary Materials

The following are available online: Figure S1: HPLC spectrum before the hydrolysis of EGb; Figure S2: Two kinds of HPLC spectra after EGb hydrolysis. (A) the HPLC spectrum of EGb extracted in this study, and (B) the HPLC spectrum of purchased EGb.

## Sample Availability

Samples of the compounds are not available from the authors.
