# Supplementary material for: Study on Synergistic Antioxidant Effect of Typical Functional Components of Hydroethanolic Leaf Extract from Ginkgo Biloba In Vitro
Source: Molecules. 2022 Jan 10;27(2):439. doi: 10.3390/molecules27020439 (PMC8778188; doi:10.3390/molecules27020439)
Supplement: Supplementary file 1 [file molecules-27-00439-s001.zip › molecules-1540040-supplementary.pdf]

Study on Synergistic Antioxidant Effect of Typical Functional Components of Hydroethanolic Leaf Extract from Ginkgo Biloba In Vitro

Lihu Zhang [1,2], Chunyi Zhu [1], Xiaoqing Liu [1], Erzheng Su [3], Fuliang Cao [4] and Linguo Zhao [1,4,*]

[1] College of Chemical Engineering, Nanjing Forestry University, Nanjing 210037, China; zlh800927@163.com (L.Z.); chunyi_zhu@163.com (C.Z.); xiaoqingliu_njfu@163.com (X.L.)
[2] Department of Pharmacy, Jiangsu Vocational College of Medicine, Yancheng 224005, China
[3] College of Light Industry Science and Engineering, Nanjing Forestry University, Nanjing 210037, China; ezhsu@njfu.edu.cn
[4] Co-Innovation Center for Sustainable Forestry in Southern China, Nanjing Forestry University, Nanjing 210037, China; caofl1953@163.com
* Correspondence: lgzhao@njfu.edu.cn; Tel.: +86-025-85428300

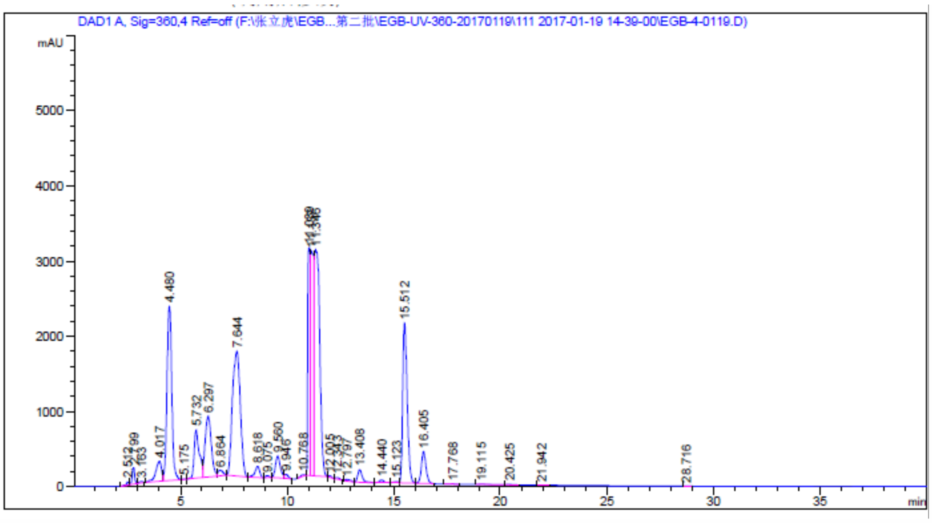

Figure S1. HPLC spectrum before the hydrolysis of EGb.

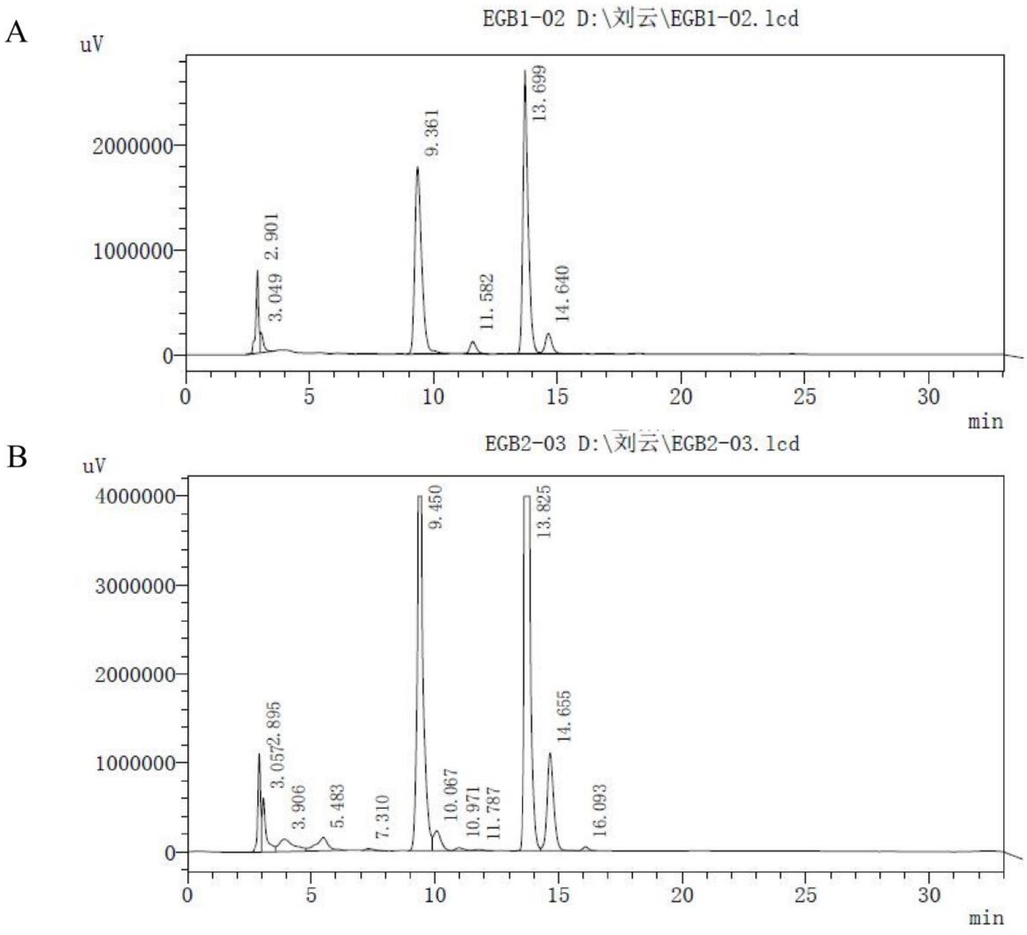

Figure S2. Two kinds of HPLC spectra after EGb hydrolysis. A) the HPLC spectrum of EGb extracted in this study, and B) the HPLC spectrum of purchased EGb.
